# Supplementary material for: Drug repurposing for aging research using model organisms
Source: Aging Cell. 2017 Jun 16;16(5):1006–15. doi: 10.1111/acel.12626 (PMC5595691; doi:10.1111/acel.12626)
Supplement: Supplementary file 7 — Data S1 Zip‐Archive of all report cards. [file ACEL-16-1006-s007.zip › RC_7PA.pdf]

7PA

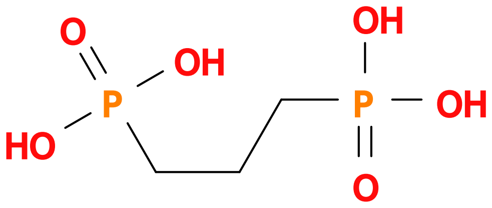

Database identifiers

ChEMBLCompound	CHEMBL1230579

Ranking

	Rank	Score
Drosophila	307/697	0.52
C. elegans	347/591	0.17

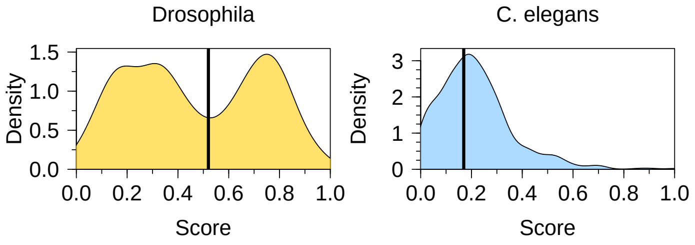

	Ageing implication	Domain conservation	Binding site conservation	Binding affinity	Bioavailability	Lipinski	Promiscuity	Purchasability	Drug approval	**Total**
Drosophila	0.792	0.933	1.0	0.783	(0.9)	0.0	-0.0	0.0	0.0	0.52
C. elegans	0.792	0.938	1.0	0.783	0.293	0.0	-0.0	0.0	0.0	0.17

Names

- 1,3-Propanediphosphonic Acid
- 3-phosphonopropylphosphonic acid
- trihydroxy-(3-trihydroxyphosphaniumylpropyl)phosphanium

Roles

ChEBI entry None has no roles

Status

Approved drug (according to ChEMBL)	No
Number of Rule of 5 violations	0
Binding affinity to original target in log units (RF-Score prediction)	6.28
Burns *C. elegans* bioavailability prediction	-0.58

Compound Target Characteristics

Thymidylate synthase

Best gene implication in ageing for this target family came from gene P45352 annotated in UniProt release 2014_02. Annotation GO 7568 (aging) was Inferred from Expression Pattern

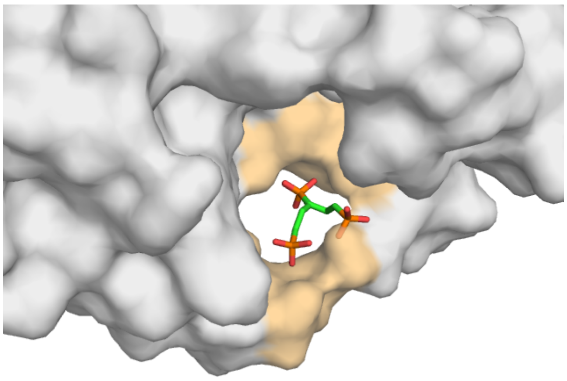

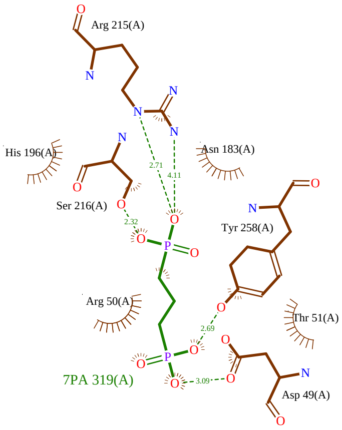

protein	amino acids contacts (binding site)
PDB:2onb:chainA:P04818	D R T N H R S Y
sp:P04818:TYSY_HUMAN	D R T N H R S Y
tr:Q53Y97:Q53Y97_HUMAN	D R T N H R S Y
sp:P45352:TYSY_RAT	D R T N H R S Y
sp:P07607:TYSY_MOUSE	D R T N H R S Y
tr:Q544L2:Q544L2_MOUSE	D R T N H R S Y
tr:Q541C9:Q541C9_DROME	D R T N H R S Y
sp:O76511:TYSY_DROME	D R T N H R S Y
tr:Q9Y052:Q9Y052_CAEEL	D R T N H R S Y
tr:Q9N588:Q9N588_CAEEL	D R T N H R S Y
sp:P06785:TYSY_YEAST	D R T N H R S Y

	whole protein		domain-based		contact-based	
protein	ident	simil	ident	simil	ident	simil
PDB:2onb:chainA:P04818	0.98	0.99	0.98	0.99	1.0	1.0
sp:P04818:TYSY_HUMAN	1.0	1.0	1.0	1.0	1.0	1.0
tr:Q53Y97:Q53Y97_HUMAN	1.0	1.0	1.0	1.0	1.0	1.0
sp:P45352:TYSY_RAT	0.88	0.95	0.92	0.98	1.0	1.0
sp:P07607:TYSY_MOUSE	0.88	0.95	0.93	0.98	1.0	1.0
tr:Q544L2:Q544L2_MOUSE	0.88	0.95	0.93	0.98	1.0	1.0
tr:Q541C9:Q541C9_DROME	0.62	0.84	0.65	0.86	1.0	1.0
sp:O76511:TYSY_DROME	0.62	0.84	0.65	0.86	1.0	1.0
tr:Q9Y052:Q9Y052_CAEEL	0.61	0.84	0.65	0.87	1.0	1.0
tr:Q9N588:Q9N588_CAEEL	0.61	0.83	0.65	0.87	1.0	1.0
sp:P06785:TYSY_YEAST	0.57	0.78	0.61	0.84	1.0	1.0

Ts (FBgn0024920) associated phenotypes

neuroanatomy defective

(Information from FlyBase)

Ts (UniProt:O76511) annotation

Pathway: Pyrimidine metabolism; dTTP biosynthesis.
Subunit: Homodimer.

(Information from UniProt)

WBGene00022455 associated phenotypes

lethal, sterile

(Information from WormBase)
